# Emotion Analysis Method of Teaching Evaluation Texts Based on Deep Learning in Big Data Environment

**DOI:** 10.1155/2022/9909209

**Published:** 2022-05-09

**Authors:** Liqin Li

**Affiliations:** Department of Preschool Education, Anyang Preschool Education College, Anyang, Henan 455000, China

## Abstract

Accurate emotion analysis of teaching evaluation texts can help teachers effectively improve the quality of education and teaching. In order to improve the precision and accuracy of emotion analysis, this paper proposes an emotion recognition and analysis method based on deep learning model. First, LTP tool is used to effectively process the teaching evaluation texts data set to improve the completeness and reliability of the data. Based on bidirectional long short-term memory (BiLSTM) network, an emotion analysis model is constructed to enhance the long-term memory ability of the model, so as to learn the emotion feature information more fully. On the basis of this model, the attention interaction mechanism module is introduced to pay attention to the important information in the attribute sequence, mine the deeper emotion feature information, and further ensure the accuracy of emotion recognition of teaching evaluation texts. Experimental simulation results show that the accuracy and precision of emotion recognition of the proposed method are 0.9123 and 0.8214, which can meet the needs of accurate emotion analysis of complex teaching evaluation texts.

## 1. Introduction

With the increase in network information resources, a large amount of text content information has been accumulated. How to make efficient use of these potential text information, mine users' satisfaction according to these text information, and then make accurate recommendations to relevant users has become the focus of more and more researchers [1, 2].

Emotion analysis refers to the process of using natural language processing technology, text analysis technology, and other related computer technology to automatically identify the emotion in subjective text and finally provide decision support [3]. It is an important branch in the field of text mining research. For colleges and universities, monitoring the curriculum quality and improving the shortcomings of curriculum teaching can effectively improve the teaching quality [4]. By introducing emotion analysis research into the process of curriculum optimization and associating the satisfaction analysis results with basic data such as teachers, courses, and departments, targeted rectification measures can be put forward and problems can be dealt with quickly, so as to effectively improve the quality of curriculum [5, 6].

College teaching evaluation data generally include structured scoring data and unstructured evaluation text data [7, 8]. At present, the research results of academic circles are mainly aimed at structured scoring data. Usually, data mining methods and statistical analysis methods are used to conduct association analysis on scoring data and other related attribute data to mine relevant knowledge [9]. At the same time, the utilization of students' teaching evaluation data in colleges and universities mostly stays in the stage of simply rewarding and punishing teachers according to the scoring results, while less attention is paid to the mining and utilization of unstructured evaluation text data.

The text data of students' teaching evaluation contain students' specific emotions and attitudes towards all aspects of teachers' teaching. Different from scoring data, text data have rich viewpoint semantic information. Deep mining of this information will help to find the satisfaction from students' perspective with teachers' teaching, so as to help teachers' improvement [10]. Therefore, it is particularly important to achieve efficient emotion analysis of teaching evaluation text data.

Traditional emotion analysis is mainly based on dictionary. The dictionary-based method mainly uses the emotion dictionary to extract the keywords expressing emotion from the corpus and then analyzes the emotion of the target sentence [11]. Reference [12] used the Chinese lexical analysis system to obtain words, gave each word an emotional tendency (positive or negative) using dictionary, and analyzed the results. Reference [13] constructed emotion classifier of public opinion texts based on Naive Bayesian theory and then judged the emotional tendency of public opinion texts in colleges and universities. The dictionary-based method can reflect the unstructured characteristics of the texts, and the classification effect is ideal when the coverage and annotation accuracy of the emotional dictionary are high [14, 15]. However, this kind of method relies on the field, language, and other background knowledge of the corpus, so it is difficult to build a high-quality emotional dictionary in practice.

With the successful application of deep learning methods in other fields, more and more deep learning methods are also applied to emotion analysis. The emotion analysis method based on deep learning can combine feature extraction and judgment of text tendency, omit the steps of manual feature extraction, and realize accurate emotion recognition research [16–18]. Reference [19] combined convolutional neural network (CNN) and recurrent neural network (RNN) to accurately extract text information and then completed text emotion analysis. Reference [20] added segmented pooling in CNN, so that the model could obtain sentence structure, extract the main features of different sentences, and analyze the emotional tendency of sentences. Reference [21] introduced convolution layer and max-pooling layer to extract local feature text and used long short-term memory network module to capture the long-term dependence between word sequences. The above references have achieved good results in automatically learning text features by using deep network, but most models do not consider the characteristics of comment text and directly carry out rough modeling without considering data such as user information and product information in the modeling process. It is difficult to realize deep mining of text information and hard to ensure the accuracy of text emotion analysis.

In view of the above problems, this paper proposes an emotion analysis method of teaching evaluation texts based on deep learning. The proposed model realizes the function of the encoder. After the attention layer, a post-LSTM layer is added to simulate the function of the decoder to extract the model features and better classify the text emotion.

## 2. Evaluation Text Data Set and Preprocessing

### 2.1. Sources of Teaching Evaluation Corpus

The corpus of students' teaching evaluation used in this paper comes from the real data of the educational administration system of H University. Since 2008, the university has implemented the teaching management activities of students' online teaching evaluation, which has rich experience in the implementation of teaching evaluation. During this period, a large number of students' teaching evaluation data have been accumulated.

H University formulates the evaluation index system from five aspects: teaching attitude, teaching content, teaching method, teaching skill, and learning harvest, and designs three different evaluation indexes of theoretical course, experimental course, and physical education course according to the nature of the course. Take the evaluation index of theory course as an example, as shown in Table 1.

When online teaching evaluation is carried out, students need to give their own value evaluation according to the real performance of teachers' classroom teaching. The value evaluation here includes two parts: objective evaluation and subjective text evaluation. Objective evaluation refers to students' Likert five-grade scoring for seven teaching evaluation indexes, respectively. Subjective text evaluation means that students give a written evaluation or suggestion of no less than 20 words for teachers' classroom teaching. For the objective scores given by students, the educational administration management system will conduct weighted summation after removing 10% of the scores before and after, and the result will be used as the final evaluation score of the student for the teacher and as one of the reward and punishment basis of the teacher. For the subjective text evaluation given by students, the administrator of the educational administration system will manually screen out some abnormal situations according to the evaluation content and reflect them to the teachers themselves, without further processing or mining the text evaluation data.

H University offers about 3000 courses every semester. At the same time, the university restricts students from participating in teaching evaluation in order to obtain the permission to select courses and view scores in the educational administration system. It makes the participation of students in online teaching evaluation of H University nearly 100%, which provides a good data basis for the research of this paper. This paper collects 126208 texts from the teaching evaluation corpus of all theoretical courses in the first semester of 2021 in the educational administration system of H University as the original corpus of this paper. At the same time, it can be seen that the students' teaching evaluation work of H University is very representative in the teaching evaluation work of college students in China. This paper selects a large number of theoretical course students' teaching evaluation text data in the university's educational administration management system for research. The research results have strong reference value and significance for the viewpoint mining of Chinese college students' teaching evaluation text data.

### 2.2. Preprocessing of Teaching Evaluation Corpus

The teaching evaluation texts are arbitrary and subjective, and the data will inevitably be mixed with some redundant comments and low value comments.

Some preprocessings of experimental data can ensure better experimental effect. In order to obtain a high-quality experimental data set, the following operations are carried out:

1. Low value comments without Chinese characters are eliminated. Without considering the comments of foreign students, comments without Chinese characters are rarely used for opinion expression, and the analysis value is not high.
2. Redundant comments of the content itself are reduced. If this operation is not carried out, the calculated emotional score will be greater than the real score of the comment, and the reduction of such comments is conducive to feature extraction.
3. The declarative comments that have no improvement suggestions and no emotional tendency are removed. Such comments have no analytical significance and have no effect on the comparison of experimental results after elimination.

In addition, the original data will contain some nonstandard words such as homophones, misspelled words, Pinyin expression, and traditional Chinese characters. Homophones, misspelled words, and traditional Chinese characters may cause the semantic dictionary to fail to match, and Pinyin expression may cause errors in text word segmentation and part of speech tagging. Therefore, these noise data need to be processed.

Text word segmentation is the process of segmenting the cleaned comment text into words by using tools or algorithms. The accuracy of word segmentation results has a significant impact on the subsequent emotional analysis. Good word segmentation results can effectively assist the completion of feature extraction tasks. If the word segmentation effect is poor, even if the subsequent algorithm is excellent, it cannot achieve good results. The current word segmentation technology has achieved great success, and the accuracy of word segmentation has been greatly improved. There are many mature word segmentation tools for developers to use. This paper uses LTP tool to segment the corpus. LTP is a natural language processing tool developed by Harbin Institute of Technology. The tool realizes rich text processing functions, such as Chinese word segmentation, part of speech tagging, and semantic dependency analysis. This paper directly uses the callable interfaces of Python and Java provided by the platform to use these functions.

The implementation of LTP word segmentation does not rely solely on the method based on dictionary matching, so the problem of too detailed word segmentation occasionally occurs. Excessive word segmentation: word segmentation is too detailed, which usually appears in four-word words. This word is a complete emotional word in teaching evaluation, but the word segmentation system divides it into two words or even four separate words. This problem is common in the existing word segmentation tools. LTP is better than other word segmentation systems in dealing with this problem, but it is still not accurate enough. To solve this problem, this paper finds that most of these words exist in the emotional dictionary. Therefore, idiom screening is carried out based on the DUTIR dictionary published by Taiwan University. The four-word words in the corpus after word segmentation are matched with the dictionary. If the matching is successful, the segmented words will be merged.

## 3. Emotion Analysis Model Based on Improved LSTM Model

### 3.1. Proposed AT-BiLSTM Model

**Figure 1:**
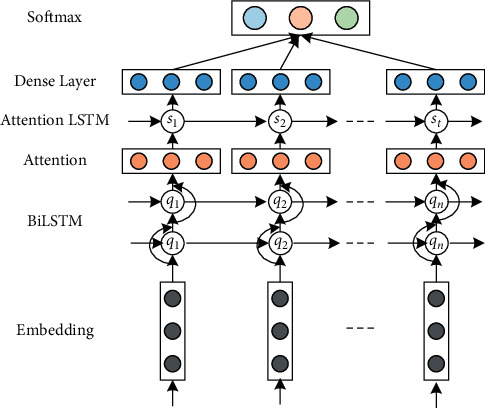
Structure of the proposed AT-BiLSTM model.

The bidirectional network is more in line with the conventional structure of the text than the unidirectional network. Therefore, the reverse LSTM is added on the basis of the original long short-term memory network, and the contextual semantic information is obtained by splicing the results of LSTM in two directions, which can better match the emotional analysis needs of the evaluation texts. As shown in Figure 1, it captures the association between the current word and other input words in the encoding stage through the bidirectional LSTM, so as to learn the more emotional feature information [22, 23]. Introducing the attention interaction mechanism into the bidirectional long short-term memory (BiLSTM) network can pay more attention to the important information in the attribute sequence, enhance the influence of keywords on sentence emotion, mine deeper emotional feature information, and further ensure the accuracy of emotional recognition of teaching evaluation texts.

### 3.2. Input Layer

According to the existing research studies, CNN can extract the morphological information in word characters (such as word prefix and suffix), and character embedding can be used as the extension of word vector. Therefore, character-level word embedding vector and word vector containing position are used as the input of the short text encoding model. The input short text word sequence is expressed as {*w*_1_, *w*_2_, *w*_3_,…, *w*_*i*_,…, *w*_*n*_}, where *w*_*i*_ represents the *i*-th word in the sentence. *w*_*i*_ word contains *j* characters, *u*_*k*_ is a vector of one character of the word *w*_*i*_, and each character represents its corresponding feature. The standard CNN is used to process the character sequence in each word and train the character-level vector of the word. The calculation formula is as follows:

(1) $$\begin{array}{c} y_{i}=\underset{1\leq i\leq j}{\max}\left(W_{CNN}\left[\begin{array}{c} y_{i}\left(w_{i\left(r-1/2\right)}\right)\\ \ldots \\ y_{i}\left(w_{i\left(r-1/2\right)}\right) \end{array}\right]+b_{CNN}\right), \end{array}$$

where *W*_*CNN*_ and *b*_*CNN*_ are the parameters of CNN and *r* is the total length of encoding model characters.

### 3.3. BiLSTM Layer

Each unit of LSTM is shown in formulas (2) to (7), where *τ* represents Sigmoid function, *g*_*t*_, *i*_*t*_,and *o*_*t*_ correspond to forgetting gate, input gate, and output gate, respectively. *p*_*t*_ is the input at time *t*, *s*_*t*_ is the unit state at time *t*, *q*_*t*_ is the LSTM output at time *t*, and *q*_*t*+1_ is the LSTM output at time *t*+1. However, LSTM can only capture the information flow in one direction, but the semantic association of text information is bidirectional. BiLSTM can obtain information from two directions, which can not only obtain more text information from the network but also more in line with the semantic characteristics of the text. BiLSTM is composed of a forward LSTM and a backward LSTM. Finally, the results of LSTM in the two directions are combined to obtain the required text features.

(2) $$\begin{array}{c} h_{t}=\tau \left(W_{h}\left[s_{t-1},p_{t}\right]+b_{h}\right), \end{array}$$

(3) $$\begin{array}{c} i_{t}=\tau \left(W_{i}\left[s_{t-1},p_{t}\right]+b_{i}\right), \end{array}$$

(4) $$\begin{array}{c} {\tilde{s}}_{t}=\tanh\left(W_{c}\left[s_{t-1},p_{t}\right]+b_{c}\right), \end{array}$$

(5) $$\begin{array}{c} s_{t}=h_{t}*s_{t-1}+i_{t}*{\tilde{s}}_{t}, \end{array}$$

(6) $$\begin{array}{c} o_{t}=\tau \left(W_{o}\left[s_{t-1},p_{t}\right]+b_{o}\right), \end{array}$$

(7) $$\begin{array}{c} q_{t}=\tau \left(W_{q}\left[s_{t-1},p_{t}\right]+b_{q}\right). \end{array}$$

As shown in formulas (8) to (10), $\overset{⟶}{L}$ and $\overset{\leftarrow }{L}$ represent LSTM in two directions and ${\overset{⟶}{q}}_{t}$ and ${\overset{\leftarrow }{q}}_{t}$ represent the output of LSTM in two directions at time *t*. *q*_*t*−1_ is the output of BiLSTM at time *t* − 1; [……,……] is a simple connection operator.

(8) $$\begin{array}{c} {\overset{⟶}{q}}_{t}=\overset{⟶}{L}\left(q_{t-1},p_{t}\right), \end{array}$$

(9) $$\begin{array}{c} {\overset{\leftarrow }{q}}_{t}=\overset{\leftarrow }{L}\left(q_{t-1},p_{t}\right), \end{array}$$

(10) $$\begin{array}{c} q_{t}=\left[{\overset{⟶}{q}}_{t},{\overset{\leftarrow }{q}}_{t}\right]. \end{array}$$

### 3.4. Attention Layer Integrating Teaching Evaluation Information

In order to extract the feature information of the text emotion database to a greater extent, this paper introduces the attention interaction mechanism into the model to make the important emotion words obtain higher weighting and realize the accurate and efficient mining of text information [24, 25].

The original attention mechanism is shown in formulas (11) and (12), where *F* is the information interaction matrix of user information *e* and text vector *q*_*t*_ and *σ*_*ij*_ is the attention of the sentence to the user. Finally, the important features in the text are screened by calculating the product of *σ*_*ij*_ and *q*_*t*_. Based on the original attention mechanism, attention interaction mechanism gradually extracts important features from user *e* to help analysis.

(11) $$\begin{array}{c} F=q_{t}\cdot e^{T}, \end{array}$$

(12) $$\begin{array}{c} \sigma_{ij}=\frac{\exp\left(F_{ij}\right)}{\sum_{i,j}\exp\left(F_{ij}\right)}. \end{array}$$

As shown in formulas (13) to (14), *δ*_*ij*_ is the user's attention to the sentence and ${\bar{\delta }}_{ij}$ is the user-level attention, that is, the important features in the filtered user information. The final attention weight *η* is obtained by the weighted sum of each user-level attention and text attention.

(13) $$\begin{array}{c} {\bar{\delta }}_{ij}=\frac{1}{n}\delta_{ij} \end{array}$$

(14) $$\begin{array}{c} \eta =\sigma \cdot {\bar{\delta }}_{ij}^{T}. \end{array}$$

As shown in formula (15), the final text representation *q*_*t*_′ is the product of the original text feature *q*_*t*_^*T*^ and the attention weight.

(15) $$\begin{array}{c} q_{t}^{\prime }=\eta \cdot q_{t}^{T}. \end{array}$$

### 3.5. Post-LSTM Layer

The background variable output by the encoder is to encode the input sequence *w*_1_, *w*_2_,…, *w*_*i*_,…, and the later attention LSTM is used as the decoder. At the time *t* of the output sequence, the decoder takes the output *q*_*t*_′ of the previous time step and the background variable as the input. It converts the hidden state *s*_*t*−1_ of the previous time step to the hidden state *s*_*t*_ of the current time step. Therefore, the conversion of the decoder hidden layer can be represented by function *z*:

(16) $$\begin{array}{c} s_{t}=z\left(q_{t-1}^{\prime },s_{t-1}\right). \end{array}$$

### 3.6. Output Layer

After the feature representation of the output of the attention layer, the emotion classification of the text can be obtained only after the processing of the classifier. For the sentence *l*, the Softmax classifier is used for classification, the predicted classification result is $\hat{j}$, and the labeled classification result is *j*, and then the calculation formula of $\hat{j}$ is as follows:

(17) $$\begin{array}{c} \hat{j}=\text{argmax}\left(\text{softmax}\left(W^{\left(l\right)}h+b^{\left(l\right)}\right)\right). \end{array}$$

The cross-entropy cost function is used as the objective function, *j* represents the emotion category of text, and $\hat{j}$ represents the predicted emotion category of text. The goal of model training is to minimize the cross-entropy of emotion categories in all sentences.

(18) $$\begin{array}{c} \text{loss}=-\sum_{m,n}j_{m}^{n}\log\;{\hat{j}}_{m}^{n}+\kappa \left\|\psi \right\|, \end{array}$$

where *m* represents the index in the sentence, *n* represents the index of the category, *κ* represents the *L*_2_ regularization, namely, the penalty term of the cost function, and *ψ* represents the set parameter. The combination of *L*_2_ regularization and Dropout is used to avoid over-fitting.

## 4. Experiments

In order to present the experimental simulation analysis with the best effect, the experiment is completed on the machine of Ubuntu 18.04.3 LTS system. At the same time, the emotion analysis network is built relying on the end-to-end open source Pytorch deep learning framework. The specific experimental platform settings are shown in Table 2.

In order to facilitate the follow-up research and analysis, the proposed model adopts the fixed parameter method. In this model, the word vector dimension of embedding layer is 300 and the output dimension of BiLSTM is 250. In the embedding layer, the value of Gaussian noise is 0.45, Dropout = 0.45, BiLSTM layer's Dropout = 0.75, L2 regularization weight = 0.00015, learning rate = 0.0008, and batch size = 55.

### 4.1. Evaluation Index

When evaluating the performance of emotion analysis, the confusion matrix method is used (Table 3). *T*_*P*_ represents the number of positive categories with correct classification as positive categories, *T*_*N*_ represents the number of negative categories with correct classification as negative categories, *F*_*P*_ represents the number of negative categories with wrong classification as positive categories, and *F*_*N*_ represents the number of positive categories with wrong classification as negative categories.

Four indexes are usually used to judge the performance of the AT-BiLSTM emotion analysis model, namely, accuracy Acc, precision Pre, recall Re, false alarm rate *F*_1_, and so on. It should be noted that for these four indexes, the higher the value, the better the detection performance.

(19) Acc=TP+TNTP+FP+TN+FN,Pre=TPTP+FP,Re=TPTP+FN,F1=2×Pre×RePre+Re.

### 4.2. Training Analysis

**Figure 2:**
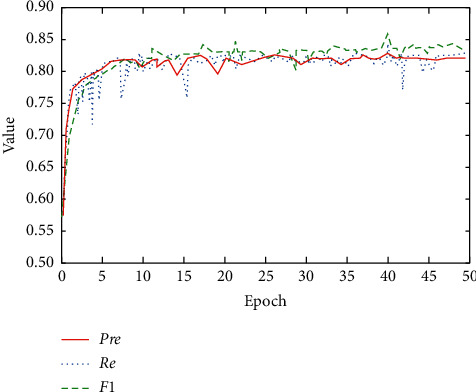
Model training and testing process.

In this paper, the teaching evaluation text data set of H University is divided into training data set and test data set according to the ratio of 4 : 1. First, the AT-BiLSTM emotion analysis model constructed in this paper is used for model learning in the training data set. During the training and testing of 50 epochs, the process diagram of precision Pre, recall Re, and false alarm rate *F*_1_ is shown in Figure 2.

**Figure 3:**
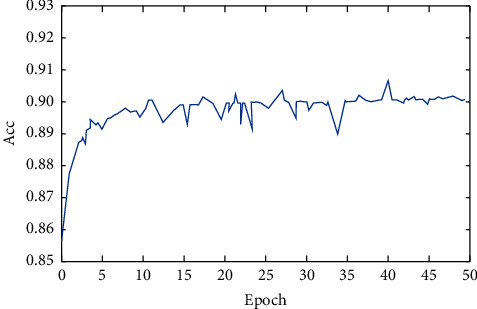
Model training accuracy.

The accuracy *Acc* obtained in the training process is shown in Figure 3.

It can be seen from Figures 2 and 3 that the proposed model obtains the optimal test result value at 40 epochs in the process of 50 epochs, as shown in Table 4.

### 4.3. Performance Comparison and Analysis of Emotion Analysis Model

**Figure 4:**
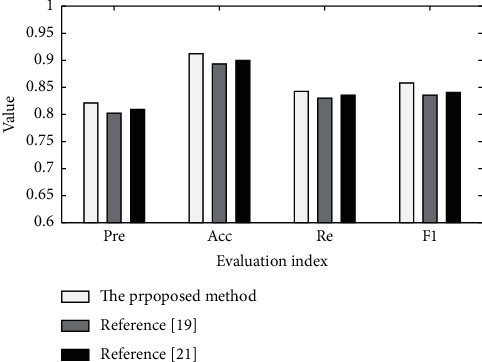
Comparison of recognition performance.

In order to verify the emotion recognition performance of the proposed method, simulation experiments are carried out comparing with the model proposed in reference [19] and reference [21] in the same environment. The results are shown in Figure 4. In order to intuitively display the emotion analysis performance of each method, the evaluation performance indexes of each method are displayed in the form of tables. It can be seen from Figure 4 and Table 5 that the proposed model can achieve more accurate text emotion analysis than the comparison methods. The accuracy *Acc* of the proposed model is 0.9123, the precision Pre is 0.8214, the recall Re is 0.8429, and the false alarm rate *F*_1_ is 0.8583. Among them, the accuracy *Acc* of the proposed model is 0.0224 higher than that in reference [19] and 0.0122 higher than that in reference [21].

The reason is that the analysis model proposed in reference [19] is a CNN network structure, which cannot distinguish the correlation between the characteristic words of the input text and emotion compared with the LSTM model, so it performs generally in the emotion analysis experiment. Reference [21] adopts the unidirectional LSTM network model, which can only capture the information flow in one direction. The unidirectional LSTM network model is contrary to the bidirectional relevance of text information semantics and does not conform to the conventional structure of text. At the same time, in order to accurately obtain the effective emotional information in the data set, the AT-BiLSTM emotional analysis model proposed in this paper introduces the attention interaction mechanism into the analysis model, further excavates the deep-seated emotional features in the sample data set, enhances the performance of the emotional analysis model, and can mine the deeper emotional feature information than references [19] and [21], so as to realize the efficient emotional recognition and analysis of the actual complex sample data set.

## 5. Conclusion

In order to improve the accuracy of emotion analysis of teaching evaluation text, this paper proposes an emotion analysis and recognition method based on the improved bidirectional LSTM model. In this method, the bidirectional LSTM model is used to construct the analysis network model, which can improve the long memory ability of the model and help to obtain the full-text information of text data. On the basis of this model, the attention interaction mechanism module is introduced to pay attention to the important information in the attribute sequence, mine the deeper emotion feature information, and further ensure the accuracy of emotion recognition of teaching evaluation texts. The simulation experiment is based on the real sample data set of H University. The simulation results show that the analysis efficiency of the proposed method is better, and the recognition accuracy reaches 0.9123, which helps to support the improvement of the quality of higher education.

Although the emotion analysis model proposed in this paper can accurately identify the actual sample data, the parameters of AT-BiLSTM model are fixed, which is difficult to automatically adjust according to the data characteristic information. The next research work is to introduce the parameter adaptive algorithm into the model to enhance the ability of parameter optimization and further improve the efficiency of emotion analysis methods.

## Figures and Tables

**Table 1:** Teaching evaluation indexes of university students.

Number	Evaluating index
1	The teacher is serious and responsible in teaching and care about the learning status of academic courses
2	The teacher is proficient in teaching clearly and easy to understand
3	The teacher's teaching content is substantial, the arrangement is reasonable, and the focus is prominent
4	The teacher uses diversified teaching methods to carry out teaching and is good at inspiring and guiding students to effectively stimulate students' interest in learning
5	The teacher pays attention to the communication and interaction with students and has good communication skills
6	The teacher is willing to answer questions both inside and outside the class
7	Through the study of this course, students feel very fruitful

**Table 2:** Settings of emotion recognition experimental analysis platform.

Project	Parameter
Operating system	Ubuntu 18.04.3 LTS
CPU	Inter(R) Core(TM) i5-7200 CPU@2.50 GHz
Graphics card	NVIDIA GeForce RTX2080TI
Memory	32 GB
Development language	Python 3.2
Development platform	Pytorch deep learning framework
Development tool	Pycharm

**Table 3:** Confusion matrix.

	True	False
True	*T*_*P*_	*T*_*N*_
False	*F*_*P*_	*F*_*N*_

**Table 4:** Optimal training indexes of the AT-BiLSTM model.

Index	Pre	*Acc*	Re	*F*_1_
Numerical value	0.8362	0.9054	0.8487	0.8619

**Table 5:** Comparison of recognition performance.

	Pre	*Acc*	Re	*F*_1_
Proposed method	0.8214	0.9123	0.8429	0.8583
Reference [19]	0.8023	0.8899	0.8301	0.8371
Reference [21]	0.8134	0.9001	0.8375	0.8435

## Data Availability

The data used to support the findings of this study are included within the article.
